# Calcineurin Signalling in Astrocytes: From Pathology to Physiology and Control of Neuronal Functions

**DOI:** 10.1007/s11064-022-03744-4

**Published:** 2022-09-09

**Authors:** Dmitry Lim, Laura Tapella, Giulia Dematteis, Maria Talmon, Armando A. Genazzani

**Affiliations:** 1 Department of Pharmaceutical Sciences, Università del Piemonte Orientale “Amedeo Avogadro”, Via Bovio 6, 28100 Novara, Italy; 2 Department of Health Sciences, School of Medicine, Università del Piemonte Orientale “Amedeo Avogadro”, Via Solaroli 17, 28100 Novara, Italy

**Keywords:** Astrocytes, Calcineurin, Proteostasis, Neuronal excitability, Memory, Epilepsy

## Abstract

Calcineurin (CaN), a Ca^2+^/calmodulin-activated serine/threonine phosphatase, acts as a Ca^2+^-sensitive switch regulating cellular functions through protein dephosphorylation and activation of gene transcription. In astrocytes, the principal homeostatic cells in the CNS, over-activation of CaN is known to drive pathological transcriptional remodelling, associated with neuroinflammation in diseases such as Alzheimer’s disease, epilepsy and brain trauma. Recent reports suggest that, in physiological conditions, the activity of CaN in astrocytes is transcription-independent and is required for maintenance of basal protein synthesis rate and activation of astrocytic Na^+^/K^+^ pump thereby contributing to neuronal functions such as neuronal excitability and memory formation. In this contribution we overview the role of Ca^2+^ and CaN signalling in astroglial pathophysiology focusing on the emerging physiological role of CaN in astrocytes. We propose a model for the context-dependent switch of CaN activity from the post-transcriptional regulation of cell proteostasis in healthy astrocytes to the CaN-dependent transcriptional activation in neuroinflammation-associated diseases.

## Introduction

Astrocytes represent a logistic arm of the CNS, assuming full homeostatic control over the CNS development and functions [1, 2]. In all these activities Ca^2+^ signals are thought to play important roles [3–6]. During the last two decades significant progress has been made in understanding the spatio-temporal properties of Ca^2+^ signals in astrocytes and their role in synaptic plasticity, memory, cognition, sleep and behaviour [3, 7–15]. This progress regarded mainly the properties and roles of Ca^2+^ signals themselves, while little was known about astrocyte-intrinsic mechanisms of decoding Ca^2+^ signals and their mechanistic link with homeostatic and signalling events in astrocytes [4]. The importance of such signalling for cell physiology can be exemplified by the role of two Ca^2+^-sensitive switches, CaMKII and CaN, in neuronal physiology, including synaptic plasticity and memory [16–18]. In astrocytes, CaMKII has been mainly studied in vitro (reviewed in [4]). CaN expression and activation in astrocytes has been detected both in vitro and in vivo and has mainly been studied in association with its downstream inflammation-related targets, transcription factors NFAT and NF-kB, in pathological conditions [19–21]. Recently, it has been suggested CaN may participate in short- and long-lasting morpho-functional changes that astrocytes undergo during neuronal activity, so called astrocytic plasticity [21]. Shortly after, evidence has been provided that CaN in astrocytes are not only implicated in the development of reactive gliosis and neuroinflammation, but have also a role in astrocytic physiology, opening to the possibility that Ca^2+^ and CaM-regulated molecular switches are important for astrocytic functions, albeit by different mechanisms [22, 23]. In this contribution we review i) astrocytic Ca^2+^ signalling in the context of CaN activation; ii) the role and mechanisms of CaN overactivation in neuropathological conditions; and iii) the mechanisms of CaN regulation of astrocytic homeostatic and signalling functions.

### Astroglial Ca^***2***+^***Signaling: An Overview***

#### Astroglial Ca^*2*+^*Signaling Toolkit*

Astrocytes do not generate action potentials and therefore are considered to be non-excitable cells, although the expression of voltage-gated ion channels in astrocytes has been demonstrated [2]. Ligand-operated signaling through ionotropic or metabotropic receptors, linked to the release of Ca^2+^ from internal stores, represents for astrocytes the main mean of signal transduction [4, 24]. Therefore, the ER membrane (endomembrane) represents an excitable media and Ca^2+^ is the substrate for the astrocytic excitability [25, 26]. Astrocytes express an array of metabotropic receptors linked to trimeric G-proteins containing Gα_q/11_, which, through activation of PLCβ, hydrolyze PIP_2_ to generate the diffusible second messenger IP_3_. IP_3_ act on IP_3_-sensitive ligand-gated channels (IP_3_Rs) located on the endomembrane allowing a controlled release of Ca^2+^ from the ER and related organelles, such as the Golgi apparatus [27–32]. IP_3_R is coded by three genes, *ITPR1-3*, resulting in three protein isoforms, IP_3_R1-3, respectively, with multiple splice variants [33]. Although all three isoforms have been found in astrocytes at mRNA level, two of them, namely IP_3_R1 and IP_3_R2 have been mainly implicated in Ca^2+^ release from the ER [14]. IP_3_R2 is thought to be the most abundant IP_3_ receptor. It mediates Ca^2+^ release from the ER in the cell soma, main and secondary astrocytic processes, named as branches and branchlets, respectively [4, 14]. Astrocyte-specific KO of IP_3_R2 abrogated the majority of massive, high amplitude Ca^2+^ signals, but not peripheral local small amplitude Ca^2+^ events. Therefore, it is thought that IP_3_R2 is responsible for the global Ca^2+^ response and for spreading of intracellular Ca^2+^ waves through the astrocyte. Instead, IP_3_R1 has been found in peripheral processes and in peri-synaptic processes, where it mediates local Ca^2+^ signals [13, 14, 34].

Ca^2+^ entry may occur through a number of ligand-gated channels which include glutamate-sensitive NMDA receptors, purinergic P2X7 receptors, dopamine D1/D2 receptors, α7-containing nicotinic acetylcholine receptors, which are expressed in a brain region-specific manner. Store-operated Ca^2+^ channels, activated in response to ER Ca^2+^ depletion, include Orai and TRP channels. STIM serves as an ER luminal Ca^2+^ sensor and transduces the change of the ER [Ca^2+^] to the plasma membrane. It has been suggested, in astrocytes, that TRPC channels (isoforms 1, 3 and 5) play a major role in SOCE [2, 4].

Ca^2+^ dynamics in astrocytes are tightly coupled with Na^+^ fluxes. The coordinating role of NCX in Ca^2+^-Na^+^ signaling interplay has been extensively discussed [35]. Functional role and Ca^2+^ entry mechanism in vivo is not completely understood and this field currently is under intensive investigation [36–38].

#### Complexity and Diversity of Astrocytic Ca^2+^ Signals

Astrocytes display a remarkable diversity of cytosolic Ca^2+^ signals, which can roughly be divided in global Ca^2+^ events and local propagating or non-propagating Ca^2+^ events. In situ and in vivo astrocytes generate frequent spontaneous Ca^2+^ signals, which occur locally in branches, branchlets and endfeet, and are independent on somatic IP_3_R2-dependent Ca^2+^ signals. Local Ca^2+^ signals may originate from (i) IP_3_R1-mediated Ca^2+^ release; (ii) Ca^2+^ influx through ligand-gated Ca^2+^ channels, TRP channels or reverse mode acting NCX; (iii) controlled Ca^2+^ efflux from mitochondria through mitochondrial permeability transition pore or from Na^+^/Ca^2+^/Li^+^ exchanger NCLX. Spontaneous Ca^2+^ events are likely to represent, or respond to the changes of metabolic or RedOx states of astrocytes. Triggered Ca^2+^ events can be evoked in response to neuronal activity as well as a part of propagating inter-cellular Ca^2+^ wave [4, 8, 12, 15, 39, 40].

Technological advances in fast volumetric imaging have allowed recording of three-dimensional Ca^2+^ signals with high speed [41]. These measurements have confirmed a rich repertoire of spontaneous and evoked Ca^2+^ signals in astrocytes, highlighting their spatio-temporal complexity and emphasizing the challenges in their detection and interpretation [12, 40, 42].

#### Functional Significance of Ca^*2*+^ Signals in Astrocytes: The State of the Art

**Fig. 1:**
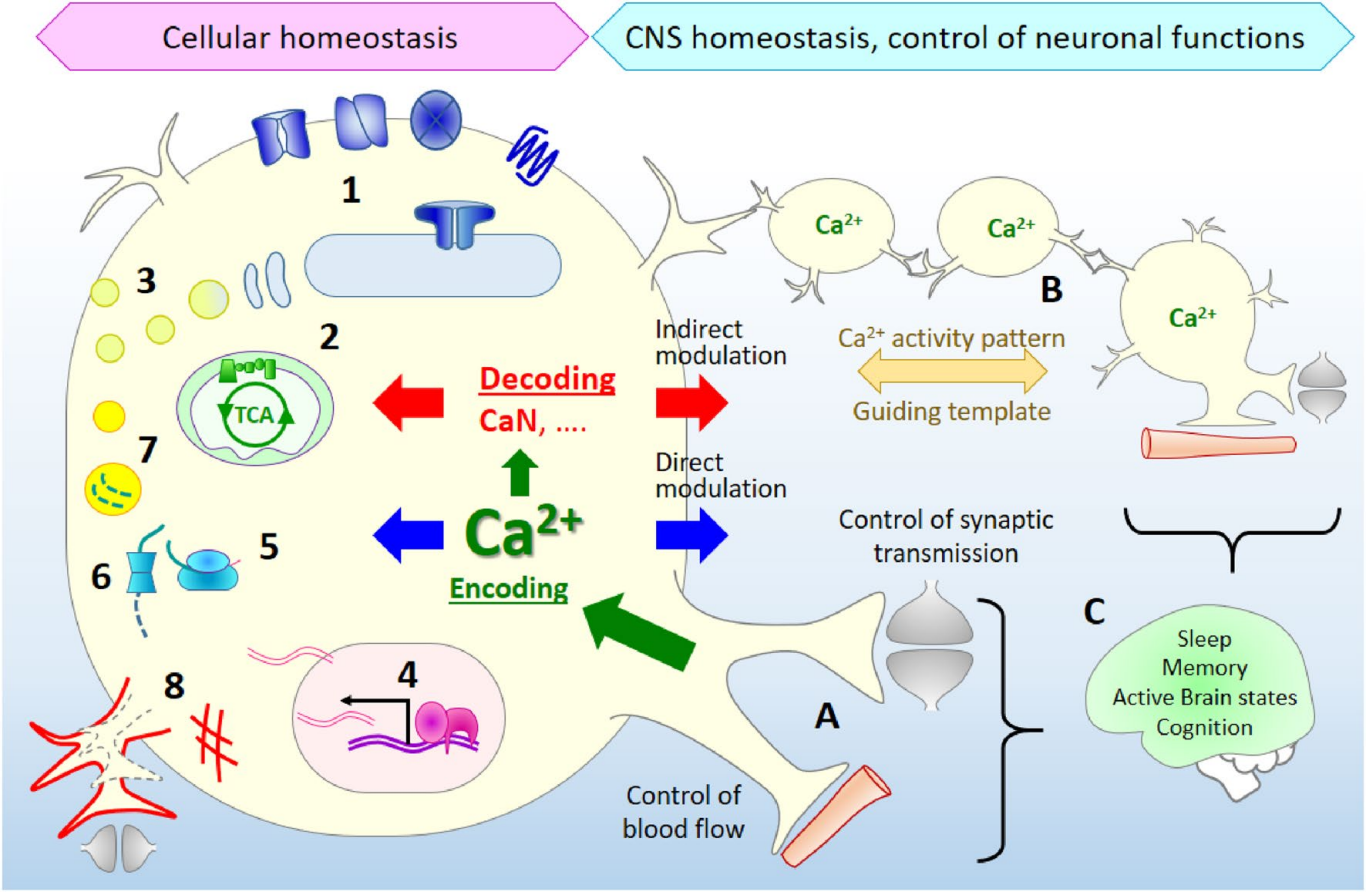
Generic and cell-specific functions of astrocytic Ca^2+^ signals. Generic Ca^2+^-regulated processes in astrocytes include: (1) regulation of Ca^2+^ homeostasis, (2) metabolism and cellular bioenergetics, (3) excitation-secretion coupling, (4) gene transcription, (5) protein synthesis, (6) proteasomal and (7) autophagosomal degradation, (8) motility and growth. Cell-specific functions of astrocytic Ca^2+^ signals classified by the complexity of the regulated functions: A modulation of synaptic transmission and regulation of local hemodynamic; B Ca^2+^ activity pattern in astrocytic syncytium and generation of guiding templates for neuronal networks; C control of systemic blood pressure and contribution to higher brain functions including respiratory control, sleep, locomotion, memory and cognition. The regulation may further be classified as (i) processes directly regulated by Ca^2+^ ions (blue arrows) like vesicular exocytosis (gliocrine function) and regulation of the activity of ion channels and transporters; (ii) indirect regulation which implicates decodification of the spatio-temporal pattern of [Ca^2+^]_i_ (red arrows and text) by calmodulin and other Ca^2+^-binding enzymes. To date, only calcineurin (CaN) has been identified in astrocytes as a Ca^2+^-sensitive molecular switch

Ca^2+^ signals are implicated in the regulation of a plethora of cellular functions and the overarching question in the field regards their functional significance [3, 4, 6]. Functionally, Ca^2+^ signaling in astrocytes can be generally divided in two categories: (1) ‘domestic’ signaling and (2) astrocyte functions-specific signaling (Fig. 1). Domestic homeostatic Ca^2+^ signaling regulates activities, common to most cells, such as (i) maintenance of Ca^2+^ homeostasis itself, (ii) secretion [43], (iii) protein synthesis and degradation [44], (iv) gene transcription [45–47], (v) proliferation and cellular motility [48, 49] and (vi) metabolism and bioenergetics [50]. Being homeostatic cells in the CNS, astrocytes use Ca^2+^ signals for sensing the environment and for translation of the environmental changes into homeostatic and signaling activities. Therefore, ‘domestic’ Ca^2+^ signaling is subordinated to the inter-cellular astrocytic responses during communication with other cells in the CNS.

Astrocyte-specific functions of Ca^2+^ signaling are thought to participate in the CNS activities at several levels, the lowest level being the control over synaptic transmission at the tripartite synapse. The next level may be represented by the control of synaptic and neuronal circuits through participation in the establishment of so called ‘guiding templates’—supra-cellular spatio-temporal patterns of signaling molecules which ‘guide’ the excitation flow in the neuronal networks. The next level of complexity may be represented by the control over systemic blood flow, respiratory control, locomotion, sleep and higher brain functions including memory formation and cognition (reviewed in [3, 12, 39, 51, 52]. All these levels have been experimentally tested for being controlled directly by astrocytic Ca^2+^ signals, mainly through the release of neuro- or vaso-active gliotransmitters and control of the ionic composition of the extracellular milieu.

However, many functions are likely to be controlled by Ca^2+^ indirectly, therefore, Ca^2+^ signals need to be ‘decoded’ and translated into modulation of protein activities, signaling events, metabolic reactions, or gene transcription events. Such Ca^2+^ ‘decoders’, represented by a limited group of Ca^2+^-binding proteins including CaMKII, CaN, Ca^2+^-activated adenylate cyclase, DREAM etc., have been studied in neurons [16, 45, 47, 53, 54], while in astrocytes there is a significant gap in knowledge. Recently, CaN has been identified as a physiologically relevant Ca^2+^ sensitive switch to drive the most important astrocytic activities [22, 55].

## Calcineurin Structure, Activation and Protein Binding

### Calcineurin Structure and Activation

CaN has been discovered in the late seventies as one of the most abundant proteins in the CNS [56, 57]. While highly enriched in neurons and in lymphoid cells, however, CaN is ubiquitously expressed in most cellular types, including astrocytes. Structurally, CaN is a heterodimer composed of one catalytic subunit (∼60 kDa, known as calcineurin A or CaNA) and one regulatory subunit (∼19 kDa, known as calcineurin B or CaNB). In mammals, three isoforms of CaNA have been identified: α, β and γ [58, 59]. The isoforms α and β are ubiquitously distributed, while the expression of γ isoform is restricted to testis. Three functional domains can be identified in the CaNA sequence: N-terminal globular catalytic domain, CaNB binding domain, and C-terminal tail containing a domain binding Ca^2+^/CaM complex (calmodulin-binding domain, CaMBD) and an autoinhibitory domain.

Two isoforms of CaNB have been identified (CaNB1 and CaNB2), with CaNB1 found to be expressed in the CNS [60]. CaNB is indispensable for the catalytic activity of CaNA, therefore its genetic deletion has been widely used to knock-out CaN in different tissues and organs. Cell-specific deletion of CaB1 has been used to ablate CaN activity in neurons and astrocytes [22, 61]. The CaNB possesses four EF-hands Ca^2+^ binding domains [62]. The first 2 EF-hands at the N-terminal lobe serve as low affinity Ca^2+^-sensing motifs, while the other 2 EF-hands at the C-terminal lobe bind Ca^2+^ with high affinity with a constant of dissociation (Kd) in the nanomolar range (30–150 nM for purified CaNB) [63]. The low affinity motifs have a regulatory role serving as sensors of the calcium fluctuations, whereas the high affinity motifs on the C-terminal lobe serve for stabilization of the heterodimer [64, 65].

In the inactive state, CaNB interacts with CaNA through N-terminal lobe, while its C-terminal lobe is loosely bound (or unbound) to the catalytic subunit. It is thought that at resting conditions the high affinity Ca^2+^-binding sites on the C-terminal lobe of CaNB are saturated with Ca^2+^ and this serves for stabilization of the CaNB-CaNA interaction. C-terminal tail of CaNA, containing CaMBD and autoinhibitory domains, is folded and interacts with CaNA catalytic site and with CaNB-binding part, impeding the interaction of substrate proteins with the catalytic site [66].

Activation of CaN has been postulated to occur in two steps. Firstly, upon elevation of cytosolic [Ca^2+^], Ca^2+^ binds to the low affinity Ca^2+^-binding sites at the N-terminal lobe of CaNB. This induces the displacement and unfolding of CaMBD and its conversion to a disordered state. This allows the interaction between the C-terminal lobe of CaNB and CaNA, and exposes the CaMBD to Ca^2+^/CaM complex. This state is considered as partially active and can be maintained in the absence of CaM. Subsequent binding of Ca^2+^/CaM complex to CaMB promotes the displacement of the autoinhibitory domain from the catalytic site of CaNA, resulting in full activation of CaN [65, 67]. Recent reports suggest that the displacement of the AID from the catalytic site requires CaM binding to the sites outside the CaMBD [68, 69].

### CaN Interaction with Proteins

Most of the knowledge on CaN binding to its substrates has been gathered studying CaN interaction with the NFAT transcription factors family [70]. Two substrate-interaction sites have been identified in the CaN holoenzyme. The PxIxIT-binding pocket is located on the side surface of the N-terminal lobe of CaNA subunit. The second site, the LxVP-binding pocket, is formed at the interface of CaNA and CaNB interaction. The PxIxIT and LxVP pockets are named after corresponding CaN-binding motifs of NFAT transcription factors family [70]. The PxIxIT pocket is accessible to CaN-binding proteins in both inactive and activated CaN states and is thought to mediate CaN-protein interaction in resting condition. Hypothetically, proteins binding to the PxIxIT pocket is possible in the absence of the regulatory CaNB subunit. In resting conditions the LxVP pocket is blocked by the interaction with CaMBD and is not accessible for CaN-binding proteins. Upon deletion of CaNB subunit, the LxVP pocket is disrupted and this leads to permanent inactivation of phosphatase CaN activity. This suggests that the substrate binding to the LxVP pocket is essential for the catalytic CaN holoenzyme activity. This conclusion is supported by the constitutive and Ca^2+^-independent CaN holoenzyme activation upon proteolytic cleavage of the C-terminal tail of CaNA containing CaMBD and AID. This also suggests that Ca^2+^ binding to CaNB is necessary exclusively for the displacement of the CaMBD and AID and does not affect catalytic activity of CaNA.

It has also been shown that the LxVP and PxIxIT motifs bind jointly to overlapping epitopes on CaNA catalytic domain distant to the regulatory domain suggesting that the ratio and the affinities of LxVP and PxIxIT motifs to CaNA define the occupancy of peptide-binding sites on CaNA [71].

During the last decade, the CaN-protein interaction field experienced a significant progress due to in silico identification of putative CaN-interacting peptides called short linear motifs (SLiMs). Hundreds of new CaN-interacting proteins have been identified containing SLiMs similar to canonical PxIxIT and LxVP motiffs and novel inhibitors of CaN signaling have been generated [72–77]. The results of these investigation suggest that: (i) binding of a protein to CaN does not automatically mean its de-phosphorylation, some CaN-binding proteins provide a scaffold for the CaN interaction with its substrates; (ii) PxIxIT- and LxVP-like motifs of CaN-binding proteins may differ from the canonical motifs, differing also in the affinity to the relative protein binding pockets on CaN; (iii) in many CaN substrates only one of two CaN binding motifs, either PxIxIT- or LxVP-like SLiM, has been identified. Considering the requirement of two-site interaction for dephosphorylation activation, this suggests the presence of yet not identified degenerated CaN-binding motifs. Otherwise, this suggests that, in certain conditions, the one-site interaction is sufficient to promote the catalysis of dephosphorylation.

Based on the above considerations and on recent reports, the following variables may define the catalytic CaN activity towards specific substrates in the intracellular environment: (i) expression levels and localization of CaNA and CaNB subunits and isoforms; (ii) spatio-temporal pattern of Ca^2+^ signals defined as a combination of spatial (global, local or in specific sub-cellular compartments) and temporal (amplitude, frequency and the duration of Ca^2+^ signals) properties [78]; (iii) intracellular distribution and concentration of CaM [79]; (iv) intracellular distribution and concentration of CaN-binding proteins, competing for binding to CaN in function of the presence and affinity of their CaN-binding motifs; (v) intracellular distribution and levels of endogenous CaN inhibitors, such as RCAN [80–82]; (vi) pathology-related constitutive activation of CaN upon cleavage by Ca^2+^-activated proteases [83, 84].

## Pathology of Astroglial Calcium and Calcineurin Signaling

Astrocytic Ca^2+^ signals have been shown to be altered in vitro and in vivo in many cell and animal models of neurodegenerative diseases with a general consensus on chronic cellular Ca^2+^ overload. In the last decade, the topic has been covered by a number of reviews and is outside the scope of this contribution [85–93]. CaN pathology in astrocytes has mainly been documented and studied in cerebro-vascular disorders, Alzheimer’s disease (AD) and brain trauma [19–21, 94, 95]. In response to chronic Ca^2+^ overload, both over-expression of CaN and its over-activation by calpain-mediated cleavage of the autoinhibitory Ca^2+^/CaM-binding tail leads to the activation of inflammation-related NFAT and NF-kB signaling. NFAT is a direct target of CaN studied in detail in immune cells [96]. In astrocytes, CaN/NFAT-mediated activation of transcriptional reprogramming towards reactive gliosis and neuroinflammation has been observed in AD models and in AD human brains [97]. In this context, the importance of astrocyte-specific CaN/NFAT signaling for the development of neuroinflammation and neuronal dysfunction, both in acute and chronic brain diseases, has been proved through molecular uncoupling of CaN from NFAT by VIVIT peptide representing a variant of CaN binding site of NFAT, optimized to bind CaN with high affinity [98–100].

There is an ample evidence of the role of NF-kB in astroglial physiology and pathology [101–103]. In astrocytes, interaction of CaN with NF-kB signaling occurs at several levels: (i) at the level of the ternary complex between Bcl10, MALT1 and CARMA1, in which Aβ-induced CaN dephosphorylation of Bcl10 positively regulates NF-kB signaling [104]; (ii) CaN dephosphorylates IkBα, thus precluding its degradation which inhibits NF-kB nuclear translocation in culture in response to anti-inflammatory action of IGF-1 [105]; (iii) CaN, in complex with NF-kB and FOXO3, is required for TNFα- induced NF-kB nuclear translocation and activation of transcription [106, 107]. The final outcome in terms of NF-kB-mediated transcriptional activation and neuroinflammatory reaction appears to be stimulus- and context-dependent, specifically, dependent on the presence of microglial cells. E.g., regulated expression of constitutively active calcineurin in astrocytes markedly reduced inflammatory injury in transgenic mice [108], while in purified cultures of hippocampal astrocytes pro-inflammatory stimuli, such as TNF, Il1β or LPS, promoted IkB degradation and NF-kB activation [109].

The therapeutic potential of CaN inhibition is exemplified by reports showing that systemic treatment of mice with CaN inhibitors mitigate neuropathology and behavior [110, 111]. Furthermore, in humans it has been shown that patients with solid organ transplants and treated with immunosuppressant CaN inhibitors for prevention of the organ rejection have a reduced risk to develop dementia [112]. This is in spite of the prior co-morbid conditions predisposing to the development of neurodegenerative diseases [112].

Taken together, the above reports suggest that overexpression and over-activation of CaN in astrocytes is required for their transition from healthy to reactive phenotype and for development of neuroinflammation.

## Physiology of Astroglial Calcineurin Signaling

Until recently little was known about physiological role of CaN in astrocytes, as CaN activation only reported in pathological conditions. A proof of principal study was published five years ago showing that CaN in astrocytes can be robustly activated in mixed astrocyte-neuron hippocampal primary cultures in response to chemical induction of LTP [23], showing that in principle, astrocytic CaN can be activated during neuronal activity. Further work on an astrocyte-specific conditional CaN KO (ACN-KO mice) showed that the spectrum of physiological activities of CaN in astrocytes differs from that in brain pathology as it does not involve transcriptional remodeling of astrocytes [22, 55].

### Control of Neuronal Excitability

It has been found that ACN-KO mice show a severe impairment of excitability of cerebellar granule cells and hippocampal pyramidal neurons. It has been suggested that this impairment is due to functional inactivation of astrocytic Na /K^+^ ATPase (NKA) and inability of astrocytes to clear K^+^ from external space resulting in prolongation of the neuronal hyperpolarization and extension of the refractory period [22]. This conclusion has been corroborated by the inhibition of neuronal excitability by NKA inhibitor ouabain. Extracellular K^+^ buffering, the most archetypal homeostatic task of the astrocytes, initially attributed to the activity of Kir4.1 channels, has been demonstrated to occur through astrocytic NKA [113, 114]. It has been shown that there is an active low affinity and high capacity K^+^ uptake by the astrocyte-specific NKA isoform, composed of α2β2 isoforms [115, 116]. These data highlight the role of astroglial NKA in K^+^ clearance [116].

Another hint that astroglial CaN is implicated in control of neuronal excitability comes from the finding that in ACN-KO astrocytes the release of glutamate was inhibited in concomitance with the upregulation of astrocyte-specific glutamate transporter GLAST [22, 117]. Astrocytic control over the ambient glutamate concentrations is important for the extrasynaptic NMDA receptor tone, which is paramount for the excitability of neuronal networks and for the balance between excitation and inhibition [118]. The deficiency of astrocytic glutamate transport leads to neuronal hyper-excitability which has been associated with epileptic seizures and pathological conditions [119, 120].

It has been found that, from 5–8 months of life, ACN-KO mice show an elevated risk to develop epileptic seizures [55]. At the age of 12 months about 50% of mice, both males and females, showed at least one seizure event. Because of the onset of Cre recombinase expression in astrocytes of ACN-KO mice, hence CaN KO, occurs very early in mouse ontogenesis (from the second postnatal week), it is unlikely that seizure are the direct effect of CaN inactivation. Rather, the several months lag between CaN KO and seizure events suggests that chronic deficiency of CaN activity in astrocytes leads to compensatory or dis-compensatory reactions which in turn result in neuronal hyper-excitability and seizures.

In summary, data suggest that the acute CaN deficiency results in neuronal hypo-excitability, while chronic CaN ablation leads to hyper-excitability. Further experiments are needed to dissect mechanistic cascade leading from CaN ablation to hyper-excitability and seizures.

### Control of Spatial Memory

**Fig. 2:**
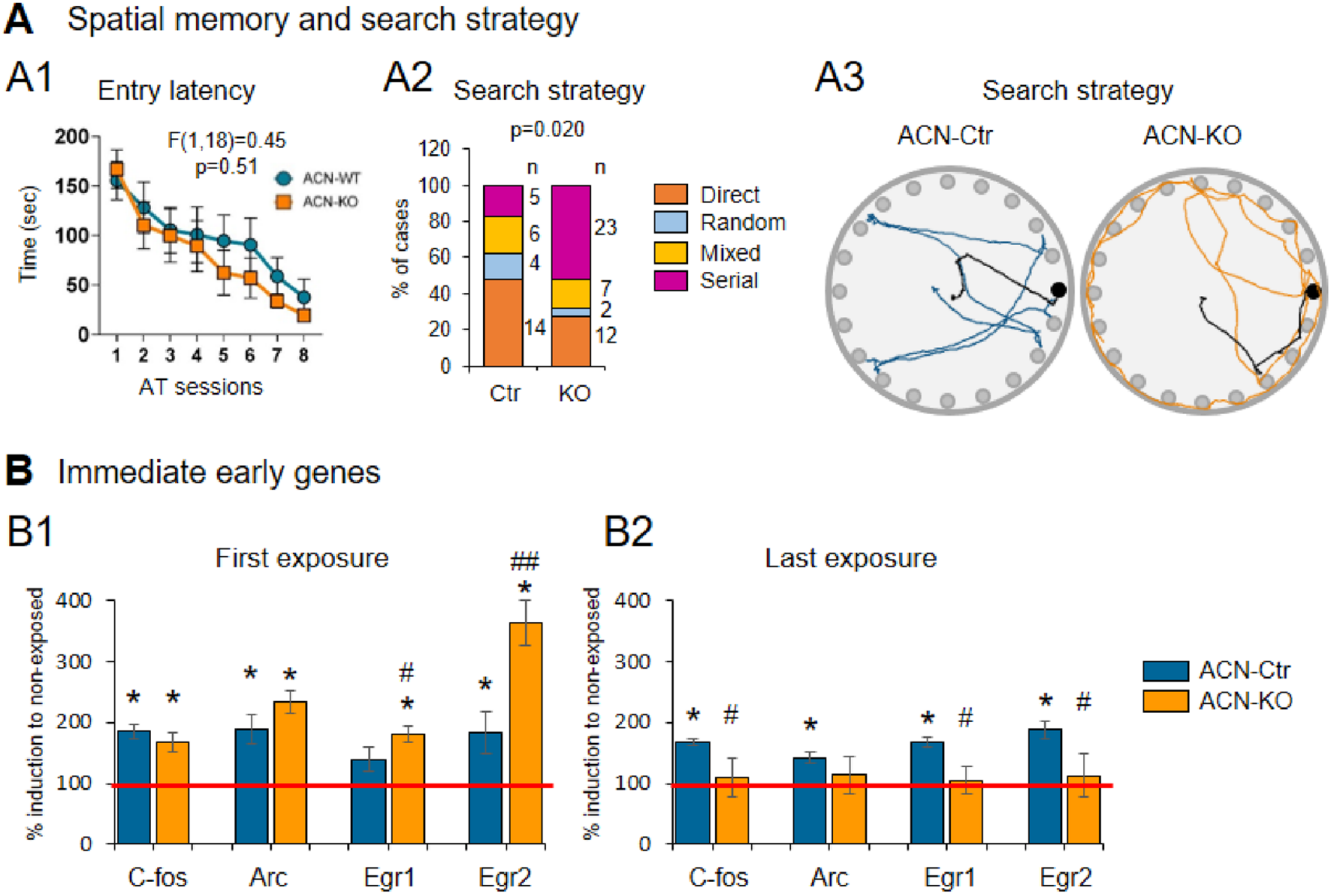
Mice with astrocyte-specific knock-out of CaNB1 learned the Barnes maze task, but use serial search strategy. Barnes maze spatial learning test was performed on 2 month-old astrocyte-specific CaNB1 knock-out mice (named as ACN-Ctr and ACN-KO mice) as described elsewhere [55]. The test consisted in one habituation session (180 s) followed by 8 acquisition training sessions (AT sessions) 90 s each during which mice were subjected to a mild intensity stress (loud sound and bright illumination). During AT sessions mice learned location of hidden escape box and the time spent to locate and enter the box (entry latency) was measured. **A**, performance of ACN-Ctr and ACN-KO mice across training sessions in Barnes maze learning paradigm. **A1**, no differences were found between entry latency learning curves suggesting that both ACN-Ctr and ACN-KO (statistical analysis was performed using 2-way ANOVA for repeated measures, F(1,18) = 0.45; p = 0.51, n = 10 mice for each genotype). **A2**, Comparison of search strategies adopted by ACN-Ctr and ACN-KO mice during acquisition trainings (Chi-square (Fisher's exact) test, p = 0.020,, n = 10 mice for each genotype). **A3**, representative traces of ACN-Ctr (blue) and ACN-KO (orange) mice of the search strategies to locate the escape box. Note that ACN-Ctr prefer direct search, while ACN-KO prefer serial search. Adapted from [55]. **B**, dorsal hippocampi were harvested either immediately after fist AT session **B1** or at the end of the last AT session **B2**, and were processed for total RNA extraction using TRIzol reagent. First cDNA strand was synthetized from 1 μg total RNA. Real-time quantitative PCR (qPCR) was performed as described elsewhere [55] using specific oligonucleotide primers for C-fos, Arc, Egr1 and Egr2 immediate early genes (IEGs). In ACN-Ctr mice, IEGs were robustly induced both at the beginning and at the end of Barnes maze test. Note higher induction of Egr1 and Egr2 IEGs after the first AT session in ACN-KO mice compared with ACN-Ctr **B1**. Strikingly, at the end of the last AT session, no induction of IEGs in ACN-KO mice was observed **B2**. *, p < 0.05 for ACN-Ctr mice exposed to compared with mice which have not been exposed to Barnes maze. #, p < 0.05 and ##, p < 0.01 for ACN-KO mice compared with ACN-KO mice for respective genes (n = 6 mice for each genotype)

Using Barnes maze paradigm of spatial memory assessment, it has been shown that ACN-KO mice do not have spatial memory impairment, i.e., they learn the position of the escape box [55]. However the way they find the box was drastically different from ACN-WT mice. ACN-WT mice use direct search using spatial queues to locate the escape box position. Instead, ACN-KO mice use serial search, which is a spatial queue-independent strategy consisting in sequential exploring holes until the right hole with the escape box is found [55]. Interestingly, the improvement of the primary latency (e.g., time to approach the hole) and the entry latency (time to enter the escape box) over the sessions was not different between ACN-WT and ACN-KO mice. i.e., ACN-KO mice remember the existence of the escape box and maintain the motivation to find it, but are likely unable to use spatial queues to locate the box (Fig. 2A). Strikingly, similar effect, i.e. use of serial instead of direct search strategy in Barnes maze learning paradigm, was reported in mice expressing Ca^2+^-insensitive mutants of CaMKII [121]. Later, it was suggested that hippocampus is used for non-spatial memory processing due to its ability to time-parse elementary memory events to integrate temporal (when), episodic (what), spatial (where) information into memory traces [122, 123]. In light of this view it is plausible to conclude that ACN-KO mice fail to develop spatial search and avail to serial exploration of holes due to functional dissociation of temporal and spatial representations both of which are hippocampus-dependent [122]. Interestingly, such a dissociation has been found in the hippocampus of an AD mouse model, rTg4510 mice, in which temporal sequences of neuronal firing can persist while spatial firing is disrupted [124]. The loss of the ability to use spatial (direct) search was found in different model of AD and epilepsy [125, 126].

While the link between astrocytic CaN and neuronal CaMKII at present is not clear, we hypothesized that, if there is a link, the expression of immediate early genes (IEGs), activated downstream CaMKII in response to the ambient stimulus, may be affected [127–129]. Indeed, we found that the induction of IEGs at the first exposure of mice to Barnes maze and at the end of training sessions, was changed oppositely: at the first exposure there is a significantly enhanced mRNA expression of Egr1 and Egr2 in ACN-KO mice compared with ACN-KO mice, while after the last session of the learning paradigm the induction of all four IEGs tested (c-Fos, Arc, Egr1 and Egr2) was completely suppressed in ACN-KO mice (Fig. 2B). While the mechanistic explanation of such effects requires further investigation, one could speculate that the mechanisms may include (i) altered mechanisms of memory consolidation which requires expression of IEGs [130]; (ii) astrocytic control of neuronal protein synthesis, discussed in the next section, and (iii) the link between neuronal excitability, found to be impaired in ACN-KO mice [22], and the activity of CaMKII in neurons [131].

### Post-Transcriptional Control of Protein Synthesis

RNA-sequencing of hippocampal and cerebellar tissues from 1 month-old ACN-KO mice showed no background astrocytic CaN-dependent transcriptional activity in the CNS [55]. Only two genes were found to be differentially expressed in hippocampus and cerebellum (Rab31 and Vapa, in hippocampus and Dsp and Rab31 in the cerebellum). This was in striking contrast to what is known about astrocytic CaN activity in pathology-related gliosis and neuroinflammation [19, 95], suggesting that, in resting condition in healthy CNS, CaN in astrocytes does not regulate gene transcription. However, it should be clarified that such transcriptional inactivity specifically regards non stimulated resting conditions. Instead, reports suggest that CaN-mediated NFAT activation and gene transcription may occur in response to neuronal activity. First, chemical activation of neuronal activity in vitro induced robust nuclear translocation of overexpressed NFAT-GFP probe into the nucleus, suggesting that neuronal activity stimulates CaN interaction with and dephosphorylation of NFAT [23]. In this frame, early report had demonstrated that in both astrocytes and pericytes of rat cortical brain slices electrical field stimulation induced CaN-dependent nuclear translocation of NFATc3 [132]. Together, these reports suggest that neuronal activity may stimulates CaN-dependent NFAT activation and, in principle, gene transcription, although functional consequences of such activation is yet to be investigated.

Further assessment of protein expression by shotgun mass spectrometry proteomics revealed massive changes in protein expression profile both in the hippocampus and cerebellum suggesting that the activity of CaN in astrocytes is directed towards regulation of posttranscriptional protein synthesis [55]. Several features make ACN-KO hippocampal and cerebellar proteomes unique with a clear association to neuropathology.

First, astrocyte-specific deletion of CaN affected protein expression in all CNS cellular types, including neurons, astrocytes, microglia and endothelial cells, with the major part of the differentially expressed proteins being neuron-specific or enriched in neurons, while there were only a few astrocyte-specific proteins. This suggests that CaN activity in astrocytes is important for the maintenance of proteostasis not only in astrocytes themselves, but in the entire CNS, which may reflect the archetypal homeostatic functions of astrocytes. Such a control may be exerted, e.g., through the control over extracellular glutamate levels [22, 117]. There is a firm association between synaptic and extra-synaptic glutamate in the CNS and protein synthesis and degradation in neurons [133–135].

Second, gene ontology analysis suggest that the major impact of CaN deletion from astrocytes is on protein synthesis and mitochondria [55]. Both disproteostasis and mitochondrial dysfunction are early alterations of many neurodegenerative and neurological diseases, and are interdependent phenomena [136, 137]. In vitro experiments on mouse and human astrocytic primary cultures suggest that inhibition of CaN results in a biphasic alteration of protein synthesis of selected proteins. Thus, GLAST was downregulated upon acute CaN inhibition, while chronic CaN inhibition results in GLAST upregulation and a reduction of extracellular glutamate [22, 117]. Moreover, CaN inhibition affected both protein synthesis and protein degradation [117]

Third, signaling pathway analysis using IPA suggested that hypothetical upstream regulators of the changes found in ACN-KO mouse hippocampus could be the AD-causing proteins MAPT, APP and PSEN and HD-causing HTT [55]. Because of the observed proteome alterations in ACN-KO mice, that are the result of the deletion of CaN from astrocytes, it is plausible to speculate that similar CaN dysfunction may occurs in neurodegenerative disease, specifically in AD. Further confirmation of this speculation is found in a significant overlap between the ACN-KO hippocampal proteome and the proteome of 5xFAD mice at early symptomatic stage: of 18 proteins found to be common in two datasets, 13 were co-regulated, 8 were co-upregulated and 5 were co-downregulated, suggesting that alterations of CaN activation in astrocytes may represent a common element [55, 138, 139].

CaN regulation of protein synthesis and degradation machineries may involve several mechanisms. At the level of ribosomal translational machinery, at least four factors, eukaryotic translation initiation factor 2B (eIF2B), eukaryotic translation initiation factor 4E binding protein 1 (4EBP1), eukaryotic translation initiation factor 4F (eIF4F) and eukaryotic translation elongation factor 2 (eEF2), have been suggested to be directly regulated by CaN [140]. Lysosomal protein degradation and autophagy have also been associated with CaN activity [141–143]. ER stress/unfolded protein response (UPR) is a stereotyped cellular reaction to stress, triggered by the accumulation of misfolded/aggregated proteins along the secretory pathway and/or by the ER Ca^2+^ dyshomeostasis [144]. There is ample evidence for the role of CaN in ER stress/UPR-associated disproteostasis [145–150].

## A Model for a Switch in CaN Activity in Astrocytes from Physiology to Pathology

**Fig. 3:**
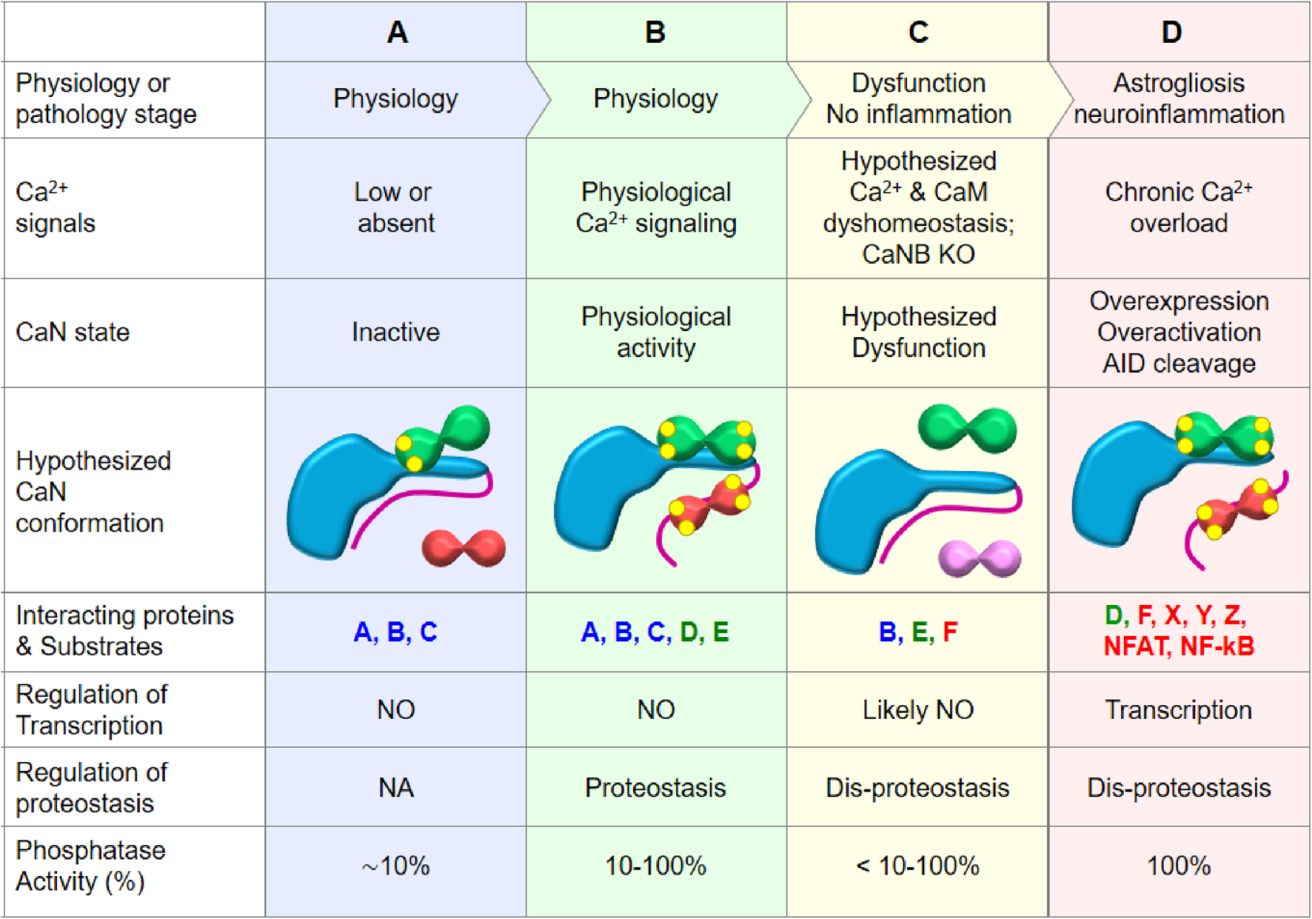
A model for CaN activity change during transition from physiological to pathological states in astrocytes. See details in the text

Based on the above discussed, we hypothesize that, in astroglial cells, the spectrum of CaN-binding partners as well as substrates for dephosphorylation may change during the transition from healthy CNS environment to pathological states, such as reactive astrogliosis-related and unrelated alterations. The model includes three main states (columns B-D in Fig. 3). First, in healthy CNS CaN in astrocytes does not interact with transcription factors, such as NFAT or NF-kB, and does not exert significant transcriptional regulation. Instead, CaN activity is required to maintain basal protein synthesis rate and degradation at the posttranscriptional level, perhaps regulating ribosomal protein synthesis and proteasomal protein degradation. The second state considers the deficiency of CaN activity due to alterations in Ca^2+^ and/or CaM signaling in response of both intrinsic and extrinsic factors which leads to disproteostasis with signatures of neuropathologies such as AD and epilepsy. Importantly, in the second state the disproteostasis does not involve activation of NFAT or NF-kB or other inflammation-related transcription factors and does not immediately result in astrocytic activation and/or neuroinflammation. This second state is based on the experiments with CaN deletion or pharmacological inhibition, it is speculative and requires experimental prove. Nevertheless, it may provide a framework for further investigation of astrocytic CaN dysfunction at early stages of neuropathologies. The third state considers the consolidated over-activation of CaN and activation of CaN → NFAT/NF-kB pathways with consequent transcriptional remodeling and development of neuroinflammation, described in a number of experimental and human pathologies including AD, epilepsy and brain trauma. This, terminal, CaN activation involves: (i) chronic overload of the cell with Ca^2+^ which generates non-physiological Ca^2+^ signal for CaN activation; (ii) overexpression of CaM, CaN subunits and substrates such as CaNA and NFAT; and (iii) CaN cleavage of Ca^2+^-activated proteases and generation of Ca^2+^ and CaM-independent constitutively active CaN species. An important implication of the proposed model is represented by the change of the CaN-binding partners during the transition from physiological to pathological states (Fig. 3). Identification of CaN-binding proteins and substrates in healthy and diseased astrocytes will be determinant for understanding the physio-pathology of astrocytic CaN signaling.

## Conclusions and Future Directions

In astrocytes, the canonical view on CaN signaling considers the activation of pro-inflammatory CaN → NFAT/NF-kB axes governing astrocytic transcriptional remodeling in pathological conditions [19, 21, 95]. Recent reports from our lab challenged this paradigm, suggesting that in healthy astrocytes transcription-independent CaN activity is required for maintenance of basal protein synthesis possibly through dephosphorylation of eIF2α, thereby regulating astrocytic and neuronal functions such as neuronal excitability and memory formation [22, 55, 117, 151]. To provide a framework for further investigation of the role and mechanisms of activation of CaN in astrocytes in physiology and pathology, we propose a model of the transition of astrocytic CaN from the transcription-independent functions in healthy CNS to the activation of CaN → NFAT/NF-kB-mediated transcriptional reprograming in diseases accompanied by neuroinflammatory reaction. The transition may include a context-dependent switch of CaN binding protein partners. To assess the validity of this model and deepen our knowledge about physiological role of astroglial CaN, further experiments are necessary addressing following issues: (i) identification of CaN-binding partners and substrates in physiological and in astrogliosis/neuroinflammation-related conditions; (ii) characterization of synaptic connectivity and plasticity in mice bearing CaN KO in astrocytes; (iii) detailed characterization, in ACN-KO mice, of functions related to maintenance of CNS homeostasis such as neurotransmitter, water and electrolyte, metabolic homeostasis; (iv) investigation of transition from transcription-inactive to transcription competent CaN states; (v) better phenotypic and mechanistic characterization of delayed alterations in mice with astrocytic CaN KO, such as epileptic seizures and concomitant neuroinflammation.

## Data Availability

This review paper contains no datasets.
